# The rise of assertive community interventions in South Africa: a randomized control trial assessing the impact of a modified assertive intervention on readmission rates; a three year follow-up

**DOI:** 10.1186/1471-244X-14-56

**Published:** 2014-02-27

**Authors:** Ulla A Botha, Liezl Koen, Ushma Galal, Esme Jordaan, Daniel JH Niehaus

**Affiliations:** 1 Department of Psychiatry, Faculty of Medicine and Health Sciences, University of Stellenbosch, PO Box 19063, Tygerberg 7505, South Africa; 2 Medical Research Council, Bellville, South Africa; 3 Department of Statistical Sciences, University of Cape Town, Rondebosch, South Africa

**Keywords:** Assertive interventions, Developing countries, Readmission rates, Days in hospital

## Abstract

**Background:**

Many countries have over the last few years incorporated mental health assertive interventions in an attempt to address the repercussions of deinstitutionalization. Recent publications have failed to duplicate the positive outcomes reported initially which has cast doubt on the future of these interventions. We previously reported on 29 patients from a developing country who completed 12 months in an assertive intervention which was a modified version of the international assertive community treatment model. We demonstrated reduction in readmission rates as well as improvements in social functioning compared to patients from the control group. The obvious question was, however, if these outcomes could be sustained for longer periods of time. This study aims to determine if modified assertive interventions in an under-resourced setting can successfully maintain reductions in hospitalizations.

**Methods:**

Patients suffering from schizophrenia who met a modified version of Weidens’ high frequency criteria were randomized into two groups. One group received a modified assertive intervention based on the international assertive community treatment model. The other group received standard care according to the model of service delivery in this region. Data was collected after 36 months, comparing readmissions and days spent in hospital.

**Results:**

The results demonstrated significant differences between the groups. Patients in the intervention group had significantly less readmissions (p = 0.007) and spent less days in hospital compared to the patients in the control group (p = 0.013).

**Conclusion:**

Modified assertive interventions may be successful in reducing readmissions and days spent in hospital in developing countries where standard care services are less comprehensive. These interventions can be tailored in such a way to meet service needs and still remain affordable and feasible within the context of an under-resourced setting.

## Background

Assertive Community Treatment (ACT) is by now a well-known approach that has been adopted in many countries [1-3]. Initially, one of the most attractive motivators for the incorporation of this approach in mental health service delivery, was its apparent success in reducing readmission rates in so-called revolving door patients. Though considered an expensive intervention, the costs were justified under the premise that inpatient costs were generally much higher. The approach has been well researched and tested over the last twenty years, with many countries reporting on a range of outcomes, such as readmission rates, patient satisfaction, degrees of symptomatology and social functioning [4,3,2,7]. Initial studies from particularly the US and Australia, reported positive outcomes in most of these areas, which prompted UK decision makers to launch 300 Assertive Outreach teams nationwide [1-3]. Though some UK studies initially demonstrated favourable outcomes, few studies reported reduction in readmission rates. A number of recent UK studies have demonstrated no benefit from ACT interventions compared with the CMHT control groups and have concluded that the approach may no longer be justifiable, considering the cost [1,8]. The discrepancy in findings by different research groups and countries has created considerable controversy [1,9,10].

Burns et al. performed a meta-regression with 64 trials in an attempt to identify factors that may contribute to outcomes. They found that a high baseline “days in hospital” (DIH) was often associated with higher reduction and that teams with high fidelity to the ACT model, also appeared to have lower readmission rates. One of the common criticisms has been that control groups have been poorly defined [1]. Clearly, “standard care” or “treatment as usual” is quite non-specific, as standard care services vary considerably within countries and even more so between countries. Several of the studies that demonstrated no significant improved outcomes, reported that the standard care services appeared to have incorporated many of the salient features of the ACT approach, such as fixed caseloads (though larger than those in ACT), home visits and assertive follow-up [11,12]. One study even reported a standard care service with a higher fidelity score than the ACT group it was being compared with [13].

Despite the apparent downfall of the ACT approach in the UK, it is still being employed with success in other countries. A recent German study in Hamburg demonstrated reduced inpatient days in patients followed-up assertively for 12 months and concluded that the intervention was more cost-effective than the standard care service it was compared with. Though outpatient costs had been higher in the intervention group, the total cost was still lower due to the significantly more expensive inpatient costs [6]. Similarly, a recent Danish study demonstrated patients receiving an assertive intervention for two years, had less substance use, better adherence to medication and were more satisfied with their treatment. In addition to this, they also reported significantly lower usage of inpatient services compared to the control group [5].

Developing countries face the same challenges of revolving door patients and bed pressures, but have the additional burden of limited resources and lack of funding to contend with. Hanlon et al. reported that only 56.5% of African countries have community-based mental health services and only 50% have existing mental health policies [14]. One of the important recommendations from this publication was the need for strengthening of specialist mental health services and further integration of mental health service with primary health service. Patel et al. called for scaling up of cost-effective community based mental-health services in middle and low-income countries citing successes in countries such as India, Chile and China, where interventions had been modified to meet the resources and needs of the community [15]. Odenwald et al. reported on such an intervention in Somalia, which offered a 10 month programme to a group of 35 outpatients with chronic psychotic disorders. The intervention was a home-based programme which incorporated psycho-education, relapse prevention and family support and was found to be cost-effective and feasible in a low-income country [16].

In South-Africa, similar attempts have been made to address the challenges in finding a cost-effective community-based initiative. As part of a provincial initiative, the Western Cape Province launched three Assertive Community Treatment (ACT) teams in 2007. The teams followed a modified version of the ACT model, particularly in terms of case loads and visit frequency. We reported that at the one year follow-up the patients who completed the intervention demonstrated significant reduction in days spent in hospital and improvements in social functioning in comparison to patients receiving the standard care service package. Though the follow-up period was only 12 months, these were the first indicators that assertive interventions could be successfully modified to meet the needs of under-resources areas without compromising the efficacy of the intervention [17]. This supports past comments by international authors [1,18] that assertive interventions may be more effective in under-resourced areas where standard care services are less comprehensive.

The important question, however, remains whether positive outcomes can be sustained over time. It is well-known that newly established services may initially have good outcomes due to staff enthusiasm and initial smaller caseloads, but that these outcomes often tail off over time as burn-out ensues and pressure rises [11].

### Aim

The purpose of this study was to determine if modified assertive interventions in an under-resourced setting can successfully maintain reductions in hospitalizations over a 36 month follow-up period.

## Methods

This study was conducted in Stikland Hospital, one of the three large state mental health hospitals in Cape Town, South Africa. The hospital, along with two others, provides inpatient services to the whole of the Western Cape Province covering a population of approximately 5 million people. The combined bed capacity for acute psychotic patients in the three hospitals is 500. The Stikland Hospital ACT team initially consisted of a full-time psychiatrist, a social worker and a chief professional nurse, but has been expanded over time. Currently the team consists of a medical officer, a social worker, three chief psychiatric nurses and a psychiatrist. The team has access to an occupational therapist, a dual diagnosis service and a PSR-based day program.

All subjects who presented for admission over an eighteen month period and who had a previously established, documented diagnosis of schizophrenia or schizo-affective disorder (DSM-IV-TR) were eligible for inclusion. To be included as high frequency users (HFUs), subjects had to fulfil the inclusion criteria, which was modified from Weiden’s HFU-criteria (see List of criteria below) to accommodate local admission patterns and ensure the appropriate service users were targeted [19]. Subjects were excluded if they had (1) a serious, unstable co-morbid medical illness that could interfere with their ability to participate in the intervention; (2) were unable to give written informed consent or (3) if another co-morbid Axis I or II diagnosis other than schizophrenia or schizo-affective disorder was the current focus of treatment.

List of criteria: Modified Weidien’s criteria for differentiation high frequency (HFU) and low frequency (LFU) schizophrenia-spectrum disorder users of psychiatric services:

General criteria

1) Schizophrenia or Schizo-affective Disorder

2 Age 18–59 years (extremes included)

3 Needs current treatment with antipsychotic

Must meet General Criteria PLUS either (A) or (B) or (C) to be included

(A) ≥3 admissions in 18 months/≥ 5 in 36 months

(B) ≥2 admissions in 12 months AND treated with clozapine

(C) ≥2 admissions in 12 months AND ≥120 days in hospital

HFUs had to fulfill General Criteria PLUS one of A; B or.

**Figure 1:**
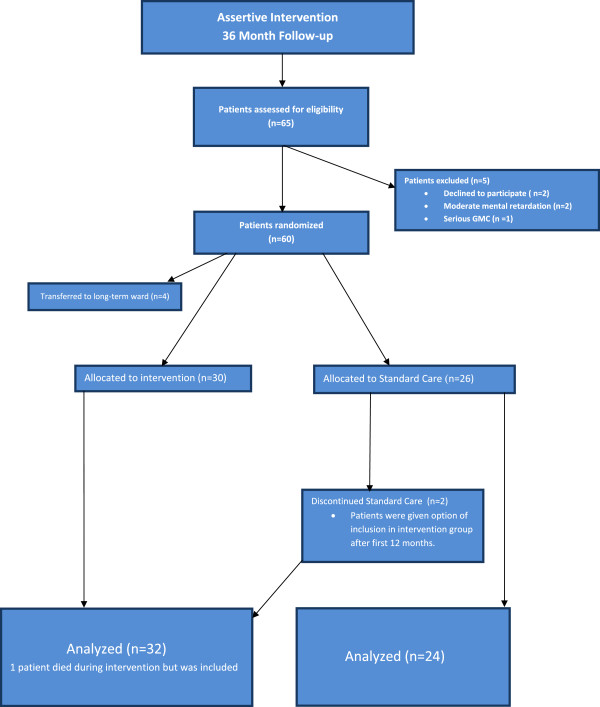
Flow diagram describing allocation of patients.

The study was approved by the research ethics committees of both the Universities of Stellenbosch and Cape Town. The research component constituted approximately half of the caseload of the ACT team whereas a non-research component provided the same intervention to high frequency users (HFUs) with other diagnoses. Research numbers therefore do not reflect overall caseloads.The trial took the form of a randomized, non-blinded parallel group study. Subjects (n = 65) identified as HFUs who provided informed, written consent, were considered for inclusion. Randomization was done using standardized tables, patients were allocated to one of two treatment groups (See Figure 1).

Subjects from both groups, a) treatment as usual (n = 26) and b) Intervention group (n = 34), received visits at inclusion, prior to discharge and after 12 months. At 36 month follow-up, data was collected from patient folders or from patients directly where no readmissions had been documented. The same method was used for both groups to obtain readmission information. Admissions data is easily accessible on the provincial data systems and also indicates admissions to other hospitals in the province. Where no readmissions were documented, patients or families were contacted to rule out any out of area admissions or adverse events. Where patients or families could not be reached, the community mental health practitioner was contacted to obtain information. Information was collected about number of readmissions, number of days spent in hospital at each admission, months in service (control or intervention respectively), number of days until first admission, number of admissions to intermediate care facility, adverse events and demographics were confirmed. Data was collected over a 72 month period; 36 months prior to date of inclusion (pre-DOI) and 36 months post -date of inclusion (post-DOI).

Subjects from the treatment as usual group were discharged into the existing standard care system. At 36 month follow-up data was collected from subject files and directly from subjects where files did not provide sufficient information.

Subjects from the intervention group were each assigned a key worker in the form of a senior social worker or a chief professional nurse. Key workers started engaging subjects and carers prior to discharge with the primary focus on building a therapeutic relationship.

The nature of the intervention was tailored as close as possible to the international model of assertive community treatment, with the two main exceptions in the size of caseloads and frequency of visits. It was agreed at the onset that the caseloads carried by international teams would not be realistic in the context of a pressured system in an under-resourced developing country. A consensus of 80 patients per team was reached, with individual caseloads not exceeding 35. Fidelity to the international model was assessed with the Dartmouth Assertive Community Treatment Scale (DACTS) with a total score of 3.1 [20]. Key workers acted as main care coordinators but caseloads were often shared between members of the team. A major focus in the team was on engagement and maintaining compliance on medication. The team also attempted to make use of existing resources in the community in addition to the service provided by the team. This may be considered a minor deviation from the ACT model where care is coordinated solely by the ACT team, but may be a practical option where teams need to spread themselves thinly. Frequency of patient contacts was individualized according to patient need with minimum of fortnightly contacts by any member of the team. Patients had access to occupational therapy and psychology services although no full-time staff was available from these disciplines. The majority of contacts (>50%) were in the community, mainly home visits.

The team was based at Stikland Hospital, one of the three state mental hospitals in the Western Cape. This had both advantages and disadvantages since the team was able to draw from the various resources in the hospital setting to strengthen the service it provided, such as access to a day programme offering psycho-social rehabilitation. The team also acted as bridge between hospital-based care and community mental health services, offering valuable liaison and streamlining communication between services. Readmissions were treated like adverse events; the team would liaise with inpatient staff and commence follow-up of patient upon discharge without any change to the follow-up period. Incarcerations were not considered in the same manner as admissions, since incarceration does not necessarily imply that appropriate psychiatric care is given. One intervention patient was incarcerated during the 36 month period but the team continued to perform visits in prison. One patient from the control group had been incarcerated during the 36 month period.

Patients in this catchment area have access to an intermediate rehabilitation facility. This unit functions as a step-up/step-down facility and offers a psycho-social rehabilitation (PSR) program. Data was collected separately on the number of patients who attended this program since this may impact on readmission rates. Patients from both groups had access to the facility but these admissions were not considered in the same way as admissions to the acute wards.

At 12 month follow-up, additional information was collected about readmissions and any changes in medication. Patients in the intervention group remained in the service and those in the control group were given the option to be included in the intervention group. Subsequently, two patients from the control group were included in the intervention group and completed a 36 month follow-up period. There was no official drop-out policy and none of the intervention patients dropped out during the first 12 months of the study. Two patients from the intervention group were discharged after 14 and 16 months of follow-up respectively. In both instances patients had been following up in the community for longer than six months without concerns about compliance or indications of relapse. Both patients had been well integrated with their respective community services and appeared no longer to require assertive input. One patient died after two months in the study. Time to readmission for this person was censored at time of death. Four patients who signed informed consent initially were referred to long-term wards (See Figure 1). Patients who were referred to long-term wards prior to their index visit were excluded. Patients who were readmitted and referred to long-term wards after a period of follow-up, were not excluded and DIH were included for analysis. Both groups were considered in the same way. Service contacts were not measured for the control group. The spectrum of standard care is very diverse and contacts are often infrequent, depending on which particular community service patients made use of. (See Table 1 for comparisons of care received between the two groups).

**Table 1:** Work style of modified ACT team compared to standard care

**Modified ACT team**		**Community mental health team**
**Overall patient load**	80-100 patients	± 600 patients excluding assessments of new patients
**Individual caseload**	Maximum 35	250
**Workstyle**	Key workers act as care coordinator but caseloads are shared	Individual caseloads
**Site of most visits**	>50% contacts are home visits	Office based
**Engagement**	Assertive; focus on engagement, immediate response to non-compliance	Non-assertive, no follow-up of missed appointments/reports of non-compliance
**Working hours**	Office hours	Office hours
**24 hour cover**	Patients referred to hospital-based after-hours service coordinated by ACT when in crisis.	After-hours service of catchment area.
**Frequency of contacts**	Individualized according to patient need at least fortnightly	Depends on caseloads, varies between monthly to three monthly.
**Disciplines available**	Full-time psychiatrist, social worker, Psychiatric nurse, access to psychologist, occupational therapist, dual diagnosis service.	Full-time psychiatric nurse, access to social worker and psychiatrist, varied access to occupational therapist and psychologist.

### Statistical analysis

Data was summarised through counts (n) and frequencies (%), medians and interquartile ranges (IQR) or means and standard deviations (sd). Wilcoxon Mann–Whitney Rank Sum tests (non-parametric) were used to test median differences while the *T*-test was used to test differences in means for normally distributed data. Fisher tests of association were used for count data.

Time to readmission was considered as the number of days to first admission after discharge, and was used as the outcome variable in the Survival analyses methods employed. Patients who completed a 36 month follow-up without readmissions were “censored” since their readmission history is not known beyond the end of the study. For Kaplan-Meier methods, data was assumed to be right-censored. To statistically test whether or not there is a difference in time to readmission between the cases and controls, and to test for covariates in the model, a Cox proportional hazards regression was carried out. The Cox regression model is a non-parametric model which assumes that the hazard rate is proportional. This assumption was tested graphically and using goodness-of-fit tests and found to be valid. Hazard ratios were calculated from the results of the regression. Since the effect of group membership on readmission was of interest, separate curves were produced so that they could be compared graphically. All statistical analyses were carried out using the package R: A Language for Data Analysis and Graphics [21].

## Results

Data were analyzed for 32 patients in the (cases) intervention group and 24 patients in the control group. The baseline demographics from both groups confirmed the homogeneity of the group for all demographic variables, except place of residence (Fisher’s test p-value = 0.01), at a 5% level of significance (Table 2). There was no missing data; all patients were successfully located at 36 month follow-up. The median age of the control group was 27.5 Years (IQR = (23.8, 36.8) years) and that for the cases was 32.0 years (IQR = (26.8, 42.8) years). To test this difference, the nonparametric Wilcoxon Mann–Whitney Rank Sum test was used and showed the difference was not statistically significant (p-value = 0.253). See Table 3 for comparisons between the two groups with regards to days spent in hospital (DIH) and number of admissions pre-and-post inclusion. When comparing days spent in hospital in the 36 months prior to inclusion (pre-DOI) in this study (Table 3), no significant difference was found between the two groups (p-value = 0.376). For the post date of inclusion data (post-DOI), there was a significant difference between the cases and controls (p-value = 0.002). To compare the pre and post-DOI data of the two groups, the difference was calculated as pre-DOI minus post-DOI number of days in hospital for each group separately. A Wilcoxon Mann–Whitney rank sum test on this data yielded a p-value of 0.013, indicating a significant difference between the two groups. However, the large confidence interval (CI = (24,177)) indicates lack of precision in the estimation, which is likely due to a patient in the control group with a long length of stay post-DOI. The Wilcoxon test was repeated without this patient and yielded a p-value of 0.023 (CI = (15, 163)). Thus, one can still conclude that there is a statistically significant difference between the pre-minus post-DOI days in hospital between the two groups.

**Table 2:** Demographic differences between cases and controls

		**Cases**	**Controls**	**Total**
**Gender**	Male	22 (69)	19 (79)	41 (73)
	Female	10 (31)	5 (21)	15 (27)
	Total:	32 (100)	24 (100)	56 (100)
**Ethnicity**	Caucasian	1 (3)	0 (0)	1 (2)
	Coloured	30 (94)	21 (88)	51 (91)
	Xhosa	1 (3)	3 (13)	4 (7)
	Total:	32 (100)	24 (100)	56 (100)
**Marital status**	Single	27 (84)	18 (75)	45 (80)
	Married	3 (9)	2 (8)	5 (9)
	Divorced	2 (6)	4 (17)	6 (11)
	Total:	32 (100)	24 (25)	56 (100)
**Language**	Afrikaans	29 (94)	21 (88)	50 (91)
	English	1 (3)	0 (0)	1 (2)
	Xhosa	1 (3)	3 (13)	4 (7)
	Total:	31 (100)	24 (100)	55 (100)
**Employment**	Unemployed	31 (97)	24 (100)	55 (98)
	Casual	1 (3)	0 (0)	1 (2)
	Total:	32 (100)	24 (100)	56 (100)
Residence**	Metro	32 (100)	19 (79)	51 (91)
	Rural	0 (0)	5 (21)	5 (9)
	Total:	32 (100)	24 (100)	56 (100)
**Accommodation**	Family	32 (100)	24 (100)	56 (100)
	Total:	32 (100)	24 (100)	56 (100)
**Highest Level of Education**	Elementary	14 (44)	5 (21)	19 (34)
	Secondary	14 (44)	14 (58)	28 (50)
	Matric	4 (13)	4 (17)	8 (14)
	None	0 (0)	1 (4)	1 (2)
	Total:	32 (100)	24 (100)	56 (100)
**Adverse events**	None	30 (94)	24 (100)	54 (96)
	Pregnancy	1 (3)	0 (0)	1 (2)
	Death	1 (3)	0 (0)	1 (2)
	Total:	32 (100)	24 (100)	56 (100)
**Status changes**	No change	27 (84)	20 (83)	47 (84)
	Discharge from Intervention	2 (6)	0 (0)	2 (4)
	Included in Intervention	2 (6)	4 (17)	6 (11)
	Death	1 (3)	0 (0)	1 (2)
	Total:	32 (100)	24 (100)	56 (100)
**Disability grant**	Yes	28 (88)	22 (92)	50 (89)
	No	4 (13)	2 (8)	6 (11)
	Total:	32 (100)	24 (100)	56 (100)

**Significant difference detected (Fisher’s test: p-value = 0.01).

**Table 3:** **Summary** - **days in hospital and number of admissions, for each group**

**Cases n = 32; controls n = 24**		**Mean (SD)**	**% of n with admissions**	
**Days in hospital**				
Pre-date of inclusion	Cases	264.8 (108.0)	100%	
	Controls	261.5 169.8)	100%	
Post-date of inclusion	Cases	35.2 (64.4)	40.60%	
	Controls	51.5 (219.2)	75%	
Pre-Post date of inclusion	Cases	229.7 (130.2)		
	Controls	110 (187.6)		
		**Median (IQR)**	**Wilcoxon test**	
			**Estimate (Cl)**	**p-value**
Pre-date of inclusion	Cases	256.0 (174.2, 319.2)	27 (−38, 92)	0.376
	Controls	202.0 (152.8, 311.5)		
Post-date of inclusion	Cases	0.0 (0.0, 52.0)	−53 (−96, −6)	0.002
	Controls	88.0 (6.8, 161.2)		
Pre-Post date of inclusion	Cases	230.0 (147.8, 314.8)	93 (24, 177)	0.013
	Controls	130.0 (57.8, 235.0)		
**Cases n = 32; controls n = 24**		**Mean (SD)**		
**Number of admissions**				
Pre-date of inclusion	Cases	4 (1.8)		
	Controls	4 (1.5)		
Post-date of inclusion	Cases	1.5 (0.8)		
	Controls	2 (1.3)		
Pre-Post date of inclusion	Cases	4 (2)		
	Controls	2 (1.8)		
		**Median (IQR)**	**Wilcoxon test**	
			**Estimate (Cl)**	**p-value**
Pre-date of inclusion	Cases	4.0 (3.0, 5.0)	0 (0, 1)	0.515
	Controls	3.0 (3.0, 4.0)		
Post-date of inclusion	Cases	0.0 (0.0, 1.0)	−1 (−2, 0)	0.001
	Controls	2.0 (0.8, 2.3)		
*Pre-Post date of inclusion	Cases	3.56 (2.0)	1.4 (0.4, 2.5)	0.007
	Controls	2.13 (1.8)		

*Summarised using mean (sd).

Table 3 also summarizes the number of readmissions for each group. The difference between the groups is again demonstrated using pre-DOI number of admissions minus post-DOI number of admissions. This data was normally distributed thus a *t*-test was used to demonstrate a difference in the means. It gave a p-value of 0.007, which indicates a significant difference in the mean pre-minus post-DOI admissions between cases and controls.

There was no significant difference between the two groups in terms of admissions to the intermediate rehabilitation facility (Fisher test p-value = 0.543). Of note was that though the number of admissions was the similar (n = 5 for controls, n = 6 for cases) in both groups, the intervention group had two patients with more than one admission to the facility, whereas all patients admitted from the control group had single admissions to the facility (See Table 4).

**Table 4:** Summary of admissions to intermediate rehabilitation facility

	**Cases**	**Controls**	**Total**
**Number of admissions to rehab facility**	**n (%)**	**n (%)**	**n (%)**
**0**	27 (84)	19 (79)	46 (82)
**1**	3 (9)	5 (21)	8 (14)
**2**	1 (3)	0 (0)	1 (2)
**3**	1 (3)	0 (0)	1 (2)
**Total**	**32 (100)**	**24 (100)**	**56 (100)**

**Figure 2:**
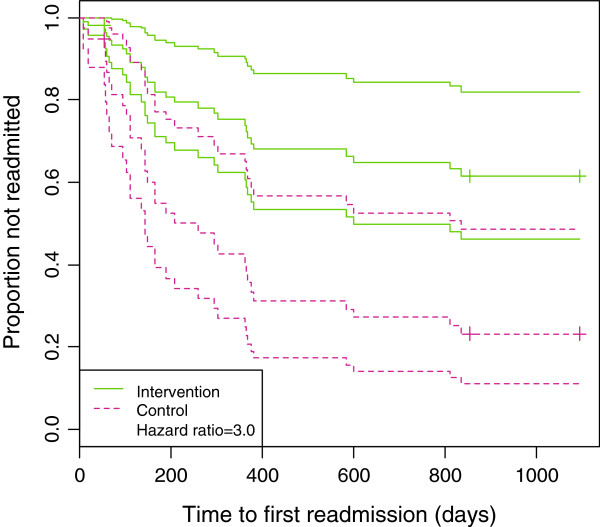
Survival curves for cases and controls from a Cox proportional hazards model.

To compare the readmission experience of cases and controls, Kaplan-Meier (K-M) Survivor Curves were plotted separately for each group (not shown). Two patients were censored; one patient died two months into the study (case) and another was included much later than other patients (control). The K-M plot clearly demonstrated that in the first 200 days there was little difference between the two group but after 200 days, the controls were more likely to be readmitted. The curves also moved further apart over time, indicating that the intervention became more beneficial over time. The K-M curves do not provide statistical evidence of a significant difference. To demonstrate this, the log-rank test was applied and gave a p-value of 0.027, which demonstrated a statistically significant difference between the readmission rates of the two groups. However, we cannot adjust for covariates using a log-rank test so a Cox Proportional Hazards model was carried out where age, gender, number of admissions pre-DOI and days in hospital pre-DOI were all adjusted for (Table 5). After adjusting for these factors, the model gave a hazard ratio of 3.0 (CI = (1.4, 6.7)), indicating a significant difference between admission experience of the cases and controls. A hazard ratio of 3.0 indicates that being in the control increases your hazard of readmission three-fold, on average. Figure 2 shows a plot of this model with separate curves for the cases and controls. Censored patients are represented by the crosses on the curves.

**Table 5:** Estimated hazard ratios from a Cox regression model

	**Hazard ratio (95% CI)**
**Group**	2.43 (1.06, 5.57)
**Gender**	1.15 (0.46, 2.84)
**Age**	0.98 (0.94, 1.03)
**Days in hospital pre-DOI**	1.00 (1.00, 1.00)
**Number of admissions pre-DOI**	1.02 (0.77, 1.35)

## Discussion

Reductions in readmission rates are widely accepted to be an effective way to assess outcome of assertive interventions and are often used as primary outcome [3]. One criticism against this method has been that this outcome may have large appeal to managers due to cost-implications and may not necessarily reflect a positive outcome for patients. However, combining this outcome with length of stay (and measurements in degrees of psychopathology) provide the necessary reassurances that the outcomes are not simply being produced by denying patients access to necessary care, but that the intervention actually reduces the need for admission [1,3,17].

The results indicate that the reduction in inpatient days that has been previously reported on, can indeed be sustained in the long run. This was considered one of the limitations of the original 12 month follow-up, since there are several factors that may contribute to short-term outcomes [17]. Both Sytema and Killaspy reported on the fact the newly established teams may initially produce positive outcomes that could tail off over time [8,11]. Newly established teams often start off with high staff enthusiasm and pressure to succeed and depending on the recruitment style, initial caseloads may be smaller than anticipated. Once caseloads increase and the novelty of the approach wears off, staff may have less time for as frequent and comprehensive input and could possibly be less likely to “go the extra mile”, so to speak. Also, since most patients were included in the service directly after an admission, one may speculate that this would reduce the number of readmissions in 12 months in itself. In addition to this, global pressures on inpatient beds mean that patients generally have to be quite ill in order to warrant admission, which in turn means that patients who are not behaviourally disturbed and pose less of a risk, may be managed in the community. These factors necessitate the use of a control group to adequately assess the effectiveness of such an intervention.

Demonstrating sustained reduction in readmissions over a 36 month period, means that the “new team” factor is less likely to contribute. By 18 months, all key workers were managing case loads of approximately 30 patients or more. In addition to this, one may speculate that having successfully demonstrated significantly reduced days in hospital, the team may have felt less pressured to sustain the level of input that had been given thus far. Surely, if “new team” enthusiasm and pressure to succeed may contribute to a teams’ ability to produce positive outcomes, the lack thereof may be expected to have the opposite effect. This was not the case however, despite the team experiencing other typical phenomenon described by established teams, such as staff-burn-out.

Days to first admission reflects the number of days from initial inclusion to first readmission. This does not necessarily reflect how stable the patients were during this time. Previous publications by our group have demonstrated higher degrees of symptomatology in the control group [17]. Clinical experience has shown that patients followed-up in a standard care setting, are seen less frequently by mental health practitioners and are often left to cope with significantly higher degrees of symptomatology prior to admission. Also, their access to inpatient services may be less streamlined than that of patients followed-up in an assertive intervention, where staff often facilitates admissions directly. This would contribute to the mean duration of stay which has been shown to be significantly longer in controls than in the intervention group. Another contributing factor in this regard, may be the fact that the particular inpatient units these patients had access to are continuously under significant pressure, with a crisis discharge policy often prompting early discharges to create beds for patient who are more ill. However, it is likely that patients who are followed-up by an intervention service may be less likely to be discharged early, since their key workers may intervene on their behalf and request that they be optimally stabilized.

It is important to note that the intervention group still had 18 readmissions. Keeping in mind that all patients met a HFU criteria at inclusion, it is inevitable that some patients will require readmission no matter how effective and comprehensive the intervention is and that the aim is not to avoid readmission at all cost. In fact, in some cases, a readmission may provide a necessary time-out for both patient and staff to revisit the treatment plan and refine the therapeutic relationship.

Though a number of recent international publications have raised serious doubts about the future of ACT teams, the approach has provided a large body of research evidence and has been vital in the development of new services [1]. While the evidence clearly indicates that benefits may be limited and cost unjustifiable in settings where standard care services are well-resourced and able to provide comprehensive care, the contrary may be true in under-resourced areas. In fact, our results indicate that even modified versions of the original approach, with significantly larger caseloads and less frequent visits, can successfully reduce inpatient usage in high frequency patients. Once again, this may reflect more on the nature of standard care than the efficacy of the intervention. Certainly there is the hope that even in under-resourced settings an approach such as this will influence the way standard care is delivered and that over time some of the salient features of the intervention will be incorporated into standard care practice, as has been the case in other settings.

## Conclusion

Assertive interventions can successfully be modified in under-resourced settings and sustain reductions in inpatient usage over time, while still remaining affordable and feasible within the context of a developing country. Such interventions need not be exclusive and limited to a small number of patients but can be successfully incorporated into existing services and tailored according to the needs of the community and resources available.

### Limitations

Single-site studies on the effectiveness of ACT tend to have small sample sizes (range 41 to 64) [6,7,11]. The reason for this may vary from country to country, but in a developing country such as RSA, limited human and financial resources are the main drivers behind this. Our sample size was limited by the small ACT team size and the limitation on caseloads (n = 80 per team member). Despite the small sample size, we were still able to demonstrate a clear advantage for ACT in terms of time to first admission and total number of re-admissions over the observation period. These findings are based on a per-protocol statistical analysis and thus only include patients who completed the treatment originally allocated. One could argue that the sample size would have been increased by including a more diverse diagnostic group. The disadvantage of such an approach is that the likelihood of unbalanced groups (in terms of diagnostic categories) will increase significantly and thus require significantly larger samples and resources. It is thus important to interpret the findings in light of limited diagnostic generalizability.

## Abbreviations

ACT: Assertive community treatment; DIH: Days in hospital; CMHT: Community mental health teams; PSR: Psycho-social rehabilitation; DSM-IV-TR: Diagnostic and statistical manual of mental disorders, Text revision; HFUs: High frequency users; DACTS: Dartmouth Assertive Community Treatment Scale; K-M: Kaplan Meier.

## Competing interests

The authors declare that they have no competing interests.

## Authors’ contributions

All authors conceived of and designed the study. UB acquired the data. UG, EJ and DN performed the statistical analysis. UB prepared the first draft of the manuscript and both LK and DN made significant contributions to the final draft. All authors read and approved the final manuscript.

## Pre-publication history

The pre-publication history for this paper can be accessed here:

http://www.biomedcentral.com/1471-244X/14/56/prepub
